# Supplementary material for: Seipin concentrates distinct neutral lipids via interactions with their acyl chain carboxyl esters
Source: J Cell Biol. 2022 Aug 8;221(9):e202112068. doi: 10.1083/jcb.202112068 (PMC9365673; doi:10.1083/jcb.202112068)
Supplement: Table S1 — list of yeast strains used in this study. [file JCB_202112068_TableS1.docx]

**Table S1 – List of yeast strains used in this study.**

| **Strain** | **Genotype** |
| --- | --- |
| yPC1505 (BY4741) | *MATa his3Δ1 leu2Δ0 met15Δ0 ura3Δ0* |
| yPC7347 | *MATa his3Δ1 leu2Δ0 met15Δ0 ura3Δ0 dga1::KAN lro1::HIS5* |
| yPC4170 | *MATa his3Δ1 leu2Δ0 met15Δ0 ura3Δ0* a*re1::NAT are2::HYGB* |
| yPC4929 | *MAT? dga1::NAT lro1::KAN are1::KAN are2::HYG* |
| yPC7813 | *MAT⍺ his3Δ1 leu2Δ0 met15Δ0 ura3Δ0 sei1::HIS* |
| yPC10794 | *MAT? his3Δ1 leu2Δ0 lys2Δ0 ura3Δ0 dga1::NAT lro1::HIS5 sei1::HYGB* |
| yPC4602 | *MAT? his3Δ1 leu2Δ0 met15Δ0 ura3Δ0 are1::KAN are2::HYG sei1::NAT* |
| yPC7930 | *MAT? his3Δ1 leu2Δ0 ura3Δ0 dga1::NAT lro1::KAN are1::KAN are2::HYGB sei1::HIS* |
| yPC4281 | *MATa his3Δ1 leu2Δ0 met15Δ0 ura3Δ0 ldb16::NAT* |
| yPC10802 | *MAT? his3Δ1 leu2Δ0 lys2Δ0 ura3Δ0 dga1::NAT lro1::HIS ldb16::HYGB* |
| yPC4645 | *MAT⍺ his3Δ1 leu2Δ0 met15Δ0 ura3Δ0 are1::KAN are2::HYGB ldb16::NAT* |
| yPC7176 | *MAT? dga1::NAT lro1::KAN are1::KAN are2::HYGB ldb16::HIS* |
| yPC4119 | *MATa his3Δ1 leu2Δ0 met15Δ0 ura3Δ0 ERG6-mCherry::URA3* |
| yPC8048 | *MATa his3Δ1 leu2Δ0 met15Δ0 ura3Δ0 nem1::* |
| yPC10629 | *MAT? his3Δ1 leu2Δ0 met15Δ0 ura3Δ0 nem1::KAN ldb16::HYGB sei1::NAT* |
| yPC10631 | *MAT? his3Δ1 leu2Δ0 met15Δ0 ura3Δ0 nem1::KAN ldb16::HYGB* |
| yPC10633 | *MAT? his3Δ1 leu2Δ0 met15Δ0 ura3Δ0 nem1::KAN sei1::NAT* |
| yPC12014 | *MATa his3Δ1 leu2Δ0 met15Δ0 ura3Δ0 dga1::NAT lro1::KAN ERG6-mCherry::URA3* |
| yPC12015 | *MATa his3Δ1 leu2Δ0 met15Δ0 ura3Δ0* a*re1::NAT are2::HYGB ERG6-mCherry::URA3* |
| yPC12016 | *MAT? dga1::NAT lro1::KAN are1::KAN are2::HYG ERG6-mCherry::URA3* |
| yPC12020 | *MATa his3Δ1 leu2Δ0 met15Δ0 ura3Δ0 SEI1-mNeonGreen::HIS3 ERG6-mCherry::URA3* |
| yPC12021 | *MATa his3Δ1 leu2Δ0 met15Δ0 ura3Δ0 dga1::NAT lro1::KAN SEI1-mNeonGreen::HIS3 ERG6-mCherry::URA3* |
| yPC12022 | *MATa his3Δ1 leu2Δ0 met15Δ0 ura3Δ0* a*re1::NAT are2::HYGB SEI1-mNeonGreen::HIS3 ERG6-mCherry::URA3* |
| yPC12023 | *MAT? dga1::NAT lro1::KAN are1::KAN are2::HYG SEI1-mNeonGreen::HIS3 ERG6-mCherry::URA3* |
| yPC12024 | *MATa his3Δ1 leu2Δ0 met15Δ0 ura3Δ0 LDB16-mNeonGreen::HIS3 ERG6-mCherry::URA3* |
| yPC12025 | *MATa his3Δ1 leu2Δ0 met15Δ0 ura3Δ0 dga1::NAT lro1::KAN LDB16-mNeonGreen::HIS3 ERG6-mCherry::URA3* |
| yPC12026 | *MATa his3Δ1 leu2Δ0 met15Δ0 ura3Δ0* a*re1::NAT are2::HYGB LDB16-mNeonGreen::HIS3 ERG6-mCherry::URA3* |
| yPC12027 | *MAT? dga1::NAT lro1::KAN are1::KAN are2::HYG LDB16-mNeonGreen::HIS3 ERG6-mCherry::URA3* |
| yPC12028 | *MATa his3Δ1 leu2Δ0 met15Δ0 ura3Δ0 SCS3-mNeonGreen::HIS3 ERG6-mCherry::URA3* |
| yPC12029 | *MATa his3Δ1 leu2Δ0 met15Δ0 ura3Δ0 dga1::NAT lro1::KAN SCS3-mNeonGreen::HIS3 ERG6-mCherry::URA3* |
| yPC12030 | *MATa his3Δ1 leu2Δ0 met15Δ0 ura3Δ0* a*re1::NAT are2::HYGB SCS3-mNeonGreen::HIS3 ERG6-mCherry::URA3* |
| yPC12031 | *MAT? dga1::NAT lro1::KAN are1::KAN are2::HYG SCS3-mNeonGreen::HIS3 ERG6-mCherry::URA3* |
| yPC12032 | *MATa his3Δ1 leu2Δ0 met15Δ0 ura3Δ0 YFT2-mNeonGreen::HIS3 ERG6-mCherry::URA3* |
| yPC12033 | *MATa his3Δ1 leu2Δ0 met15Δ0 ura3Δ0 dga1::NAT lro1::KAN YFT2-mNeonGreen::HIS3 ERG6-mCherry::URA3* |
| yPC12034 | *MATa his3Δ1 leu2Δ0 met15Δ0 ura3Δ0* a*re1::NAT are2::HYGB YFT2-mNeonGreen::HIS3 ERG6-mCherry::URA3* |
| yPC12035 | *MAT? dga1::NAT lro1::KAN are1::KAN are2::HYG YFT2-mNeonGreen::HIS3 ERG6-mCherry::URA3* |
| yPC12036 | *MATa his3Δ1 leu2Δ0 met15Δ0 ura3Δ0 PEX30-mNeonGreen::HIS3 ERG6-mCherry::URA3* |
| yPC12037 | *MATa his3Δ1 leu2Δ0 met15Δ0 ura3Δ0 dga1::NAT lro1::KAN PEX30-mNeonGreen::HIS3 ERG6-mCherry::URA3* |
| yPC12038 | *MATa his3Δ1 leu2Δ0 met15Δ0 ura3Δ0* a*re1::NAT are2::HYGB PEX30-mNeonGreen::HIS3 ERG6-mCherry::URA3* |
| yPC12039 | *MAT? dga1::NAT lro1::KAN are1::KAN are2::HYG PEX30-mNeonGreen::HIS3 ERG6-mCherry::URA3* |
| yPC4299 | *MATa his3Δ1 leu2Δ0 met15Δ0 ura3Δ0 sei1::NAT ldb16::HYG* |
| yPC12412 | *MATa his3Δ1 leu2Δ0 met15Δ0 ura3Δ0 dga1::NAT lro1::KAN sei1::HIS ldb16::HYG* |
| yPC12413 | *MAT⍺ his3Δ1 leu2Δ0 met15Δ0 ura3Δ0 are1::KAN are2::HYG sei1::HIS ldb16::NAT* |
| yPC12416 | MAT? *dga1::NAT lro1::KAN are1::KAN are2::ZEO sei1::HYG ldb16::HIS* |
