# Supplementary material for: Seipin concentrates distinct neutral lipids via interactions with their acyl chain carboxyl esters
Source: J Cell Biol. 2022 Aug 8;221(9):e202112068. doi: 10.1083/jcb.202112068 (PMC9365673; doi:10.1083/jcb.202112068)
Supplement: Table S2 — list of plasmids used in this study. [file JCB_202112068_TableS2.docx]

**Table S2 – List of plasmids used in this study.**

| **Plasmid** |  |
| --- | --- |
| B1820 | pRS316 |
| B2800 | pRS416 |
| PB563 | pRS415 *P_GAL1_* |
| pPC1125 | pRS415 *P_GAL1_-DGA1* |
| pPC1126 | pRS415 *P_GAL1_-ARE1* |
| pPC1127 | pRS415 *P_GAL1_-LRO1* |
| pPC1128 | pRS415 *P_GAL1_-ARE2* |
| pPC2124 | pRS316 *P_LDB16_-LDB16-3xFLAG* |
| pPC2130 | pRS316 *P_LDB16_-LDB16(S53/55/62A T52/61/63A)-3xFLAG* |
| bPC2121 | pRS416 *P_ADH1_-Hs_Seipin_iso1-3xFLAG* |
| bPC2122 | pRS416 *P_ADH1_-Hs_Seipin_iso1 (S165/166A)-3xFLAG* |
| bPC2123 | pRS416 *P_ADH1_-Hs_Seipin_iso1 (S165/166D)-3xFLAG* |
| pPC2159 | pRS415 *P_GAL1_-LRAT-GFP* |
