# Supplementary material for: Seipin concentrates distinct neutral lipids via interactions with their acyl chain carboxyl esters
Source: J Cell Biol. 2022 Aug 8;221(9):e202112068. doi: 10.1083/jcb.202112068 (PMC9365673; doi:10.1083/jcb.202112068)
Supplement: Table S3 — shows coarse grained MD simulations run in this study. [file JCB_202112068_TableS3.docx]

| **System** | **Simulations** |
| --- | --- |
| Hs_Seipin + complex membrane + 1% TAG + 3% SE | 3 x 5 µs |
| Hs_Seipin + complex membrane + 3% DAG + 3% SE | 3 x 5 µs |
| Hs_Seipin + complex membrane + 3% DAG + 3% RE | 3 x 5 µs |
| Hs_Seipin + complex membrane + 3% DAG + 3% SQL | 3 x 5 µs |
| Hs_Seipin + complex membrane + 1% TAG + 3% SQL | 3 x 5 µs |
| Hs_Seipin + complex membrane + 3% DAG + 3% FFA | 3 x 5 µs |
| Hs_Seipin + complex membrane + 1% TAG + 3% FFA | 3 x 5 µs |
| Hs_Seipin + complex membrane + 1% TAG + 3% DAG | 3 x 5 µs |
| POPC + 2% TAG | 1 x 5 µs |
| POPC + 3% TAG | 1 x 5 µs |
| POPC + 5% TAG | 1 x 5 µs |
| POPC + 10% TAG | 1 x 5 µs |
| POPC + 15% TAG | 1 x 5 µs |
| POPC + 20% TAG | 1 x 5 µs |
| POPC + 25% TAG | 1 x 5 µs |
| POPC + 30% TAG | 1 x 5 µs |
| POPC + 2.5% SE | 1 x 5 µs |
| POPC + 5% SE | 1 x 5 µs |
| POPC + 8% SE | 1 x 5 µs |
| POPC + 10% SE | 1 x 5 µs |
| POPC + 15% SE | 1 x 5 µs |
| POPC + 20% SE | 1 x 5 µs |
| POPC + 25% SE | 1 x 5 µs |
| POPC + 30% SE | 1 x 5 µs |
| POPC + 3% SQL | 1 x 5 µs |
| POPC + 5% SQL | 1 x 5 µs |
| POPC + 8% SQL | 1 x 5 µs |
| POPC + 10% SQL | 1 x 5 µs |
| POPC + 15% SQL | 1 x 5 µs |
| POPC + 20% SQL | 1 x 5 µs |
| POPC + 25% SQL | 1 x 5 µs |
| POPC + 30% SQL | 1 x 5 µs |
| POPC + 3% RE | 1 x 5 µs |
| POPC + 5% RE | 1 x 5 µs |
| POPC + 8% RE | 1 x 5 µs |
| POPC + 10% RE | 1 x 5 µs |
| POPC + 15% RE | 1 x 5 µs |
| POPC + 20% RE | 1 x 5 µs |
| POPC + 25% RE | 1 x 5 µs |
| POPC + 30% RE | 1 x 5 µs |

**Table S3 - Coarse grained MD simulations run in this study**

The complex membrane comprised of ca. 36% *sn*-1-palmitoyl-*sn*-2-oleoyl-phosphatidylcholine (PC), 18% *sn*-1-palmitoyl-*sn*-2-oleoyl-phosphatidylethanolamine (PE), 18% *sn*-1-palmitoyl-*sn*-2-oleoyl-phosphatidylinositol (PI), 6% *sn*-1-palmitoyl-*sn*-2-oleoyl phosphatidylserine (POPS), 6% *sn*-1-palmitoyl-*sn*-2-oleoyl-phosphatidic acid (POPA), 3% *N*-stearoyl-*D*-erythro-sphingosine (Cer), and 10% cholesterol.

Neutral lipids used are trioleoylglycerol (TAG), cholesteryl oleate (SE), retinyl palmitate (RE), *sn*-1-palmitoyl-*sn*-2-oleoyl-diacylglycerol (DAG), squalene (SQL) and oleate (FFA).
