# Supplementary figures and images for: Seipin concentrates distinct neutral lipids via interactions with their acyl chain carboxyl esters
Source: J Cell Biol. 2022 Aug 8;221(9):e202112068. doi: 10.1083/jcb.202112068 (PMC9365673; doi:10.1083/jcb.202112068)

IB: Pln1

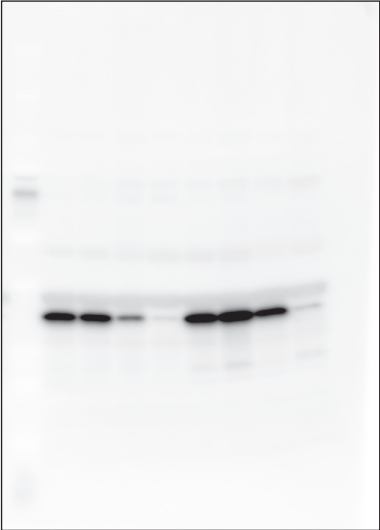

Marker (epi)

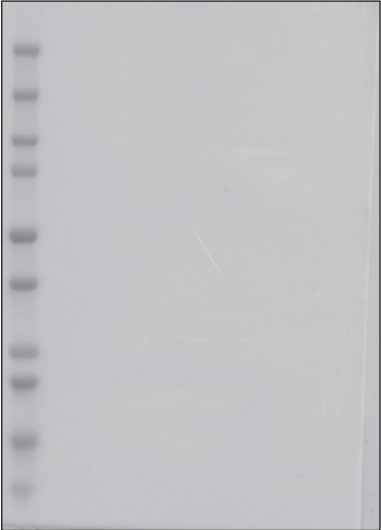

50

IB: Dpm1

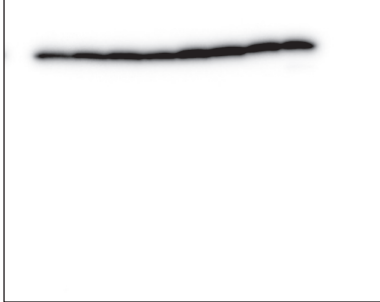

Marker (epi)

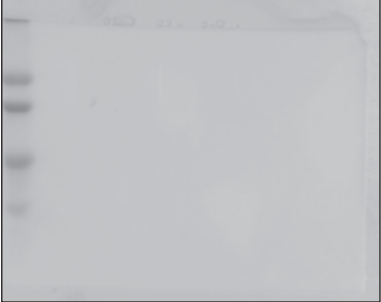

37

25

Supplement: SourceData FS1 — is the source file for Fig. S1. [file JCB_202112068_SourceDataFS1.pdf]

IB: FLAG

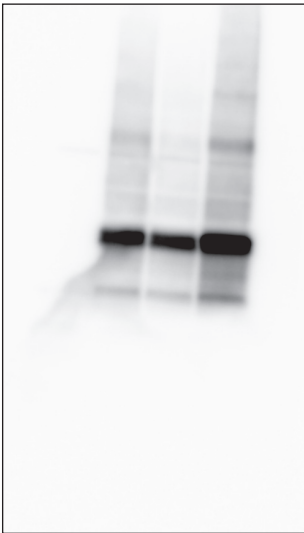

Marker (epi)

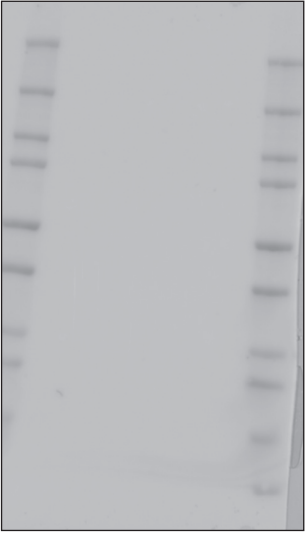

50  
37  
25

IB: Dpm1

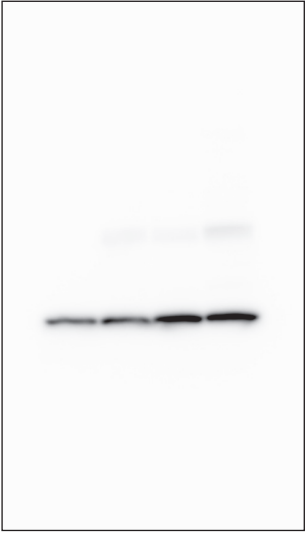

Marker (epi)

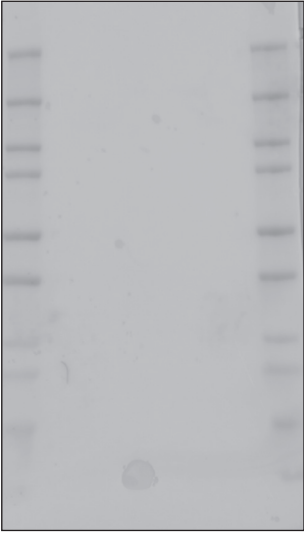

50  
37  
25

Supplement: SourceData FS4 — is the source file for Fig. S4. [file JCB_202112068_SourceDataFS4.pdf]
